# Supplementary material for: Brain–phenotype models fail for individuals who defy sample stereotypes
Source: Nature. 2022 Aug 24;609(7925):109–18. doi: 10.1038/s41586-022-05118-w (PMC9433326; doi:10.1038/s41586-022-05118-w)
Supplement: Supplementary file 1 — This file contains Supplementary Figs. 1–4, Supplementary Tables 1–11, Supplementary Discussion and Supplementary References. [file 41586_2022_5118_MOESM1_ESM.pdf]

nature portfolio

https://doi.org/10.1038/s41586-022-05118-w

Supplementary information

Brain–phenotype models fail for individuals who defy sample stereotypes

In the format provided by the authors and unedited

Supplementary Information

Brain-phenotype models fail for individuals who defy sample stereotypes

Authors: Abigail S. Greene[1,2*], Xilin Shen[3], Stephanie Noble[3], Corey Horien[1,2], C. Alice Hahn[3], Jagriti Arora[3], Fuyuze Tokoglu[3], Marisa N. Spann[4], Carmen I. Carrión[5], Daniel S. Barron[6,7,8,9], Gerard Sanacora[7], Vinod H. Srihari[7], Scott W. Woods[7], Dustin Scheinost[1,3,10,11,12], R. Todd Constable[1,3,10,13*]

[1]Interdepartmental Neuroscience Program, Yale School of Medicine; [2]MD/PhD program, Yale School of Medicine; [3]Dept. of Radiology and Biomedical Imaging, Yale School of Medicine; [4]Dept. of Psychiatry, Columbia University Irving Medical Center; [5]Dept. of Neurology, Yale School of Medicine; [6]Dept. of Anesthesiology & Pain Medicine, University of Washington; [7]Dept. of Psychiatry, Yale School of Medicine; [8]Dept. of Psychiatry, Brigham and Women's Hospital, Harvard Medical School; [9]Dept. of Anesthesiology, Perioperative and Pain Medicine, Brigham and Women's Hospital, Harvard Medical School; [10]Dept. of Biomedical Engineering, Yale School of Engineering and Applied Science; [11]Dept. of Statistics and Data Science, Yale University; [12]Child Study Center, Yale School of Medicine; [13]Dept of Neurosurgery, Yale School of Medicine

*Corresponding authors: Abigail Greene and R. Todd Constable
abigail.greene@yale.edu, todd.constable@yale.edu
Yale University
The Anlyan Center
300 Cedar Street
New Haven, CT 06520
203.785.6199

Table of Contents

Supplementary Figures 4

Supplementary Figure 1. Similarity (rank correlation) of misclassification frequency (MF; averaged across in-scanner conditions) derived from classification analyses of the given Yale phenotypic measure using raw and motion-regressed functional connectivity (FC). 4

Supplementary Figure 2. Relationships between each pair of covariates in the Yale dataset. 5

Supplementary Figure 3. Relationships between each pair of covariates in the UCLA dataset. 6

Supplementary Figure 4. Relationships between each pair of covariates in the HCP dataset. 7

Supplementary Tables 8

Supplementary Table 1. In-scanner tasks and corresponding RDoC domains and constructs. 8

Supplementary Table 2. Measures used in the post-scan behavioral battery, with corresponding RDoC domains and constructs where relevant. 9

Supplementary Table 3. Demographic and clinical information for the Yale, UCLA, and HCP datasets. 11

Supplementary Table 4. Mean classification accuracy (averaged across 100 iterations) for each phenotypic measure using FC calculated from all in-scanner conditions in the Yale dataset. 12

Supplementary Table 5. Mean classification accuracy (averaged across 100 iterations) for each phenotypic measure using FC calculated from all in-scanner conditions in the UCLA dataset. 13

Supplementary Table 6. Mean classification accuracy (averaged across 1000 iterations, given 10-fold analysis) for each phenotypic measure using FC calculated from all in-scanner conditions in the HCP dataset. 14

Supplementary Table 7. Mean classification accuracy, averaged across iterations and in-scanner conditions for each phenotypic measure in the Yale dataset, using motion-regressed (MoR) and raw FC for classification. 15

Supplementary Table 8. FDR-adjusted (90 tests) *P* values for above-chance performance in cross-dataset analyses, calculated via one-tailed comparison to distribution of accuracy across 100 iterations of classification of permuted labels. 16

Supplementary Table 9. Relationships (two-tailed rank correlation for continuous covariates; two-tailed Mann-Whitney U test for binary covariates) between covariates and mean MF. 17

Supplementary Table 10. Relationships (two-tailed rank correlation for continuous covariates; two-tailed Mann-Whitney U test for binary covariates) between covariates and mean phenotypic scores. 19

Supplementary Table 11. Relationships between continuous model fit and covariates in the Yale dataset. 21

Supplementary Discussion 22
Model failure as a tool for subtyping 22
Covariate-outcome relationships may be varied and complex 23
Additional limitations and future directions 23
Supplementary References 25

Supplementary Figures

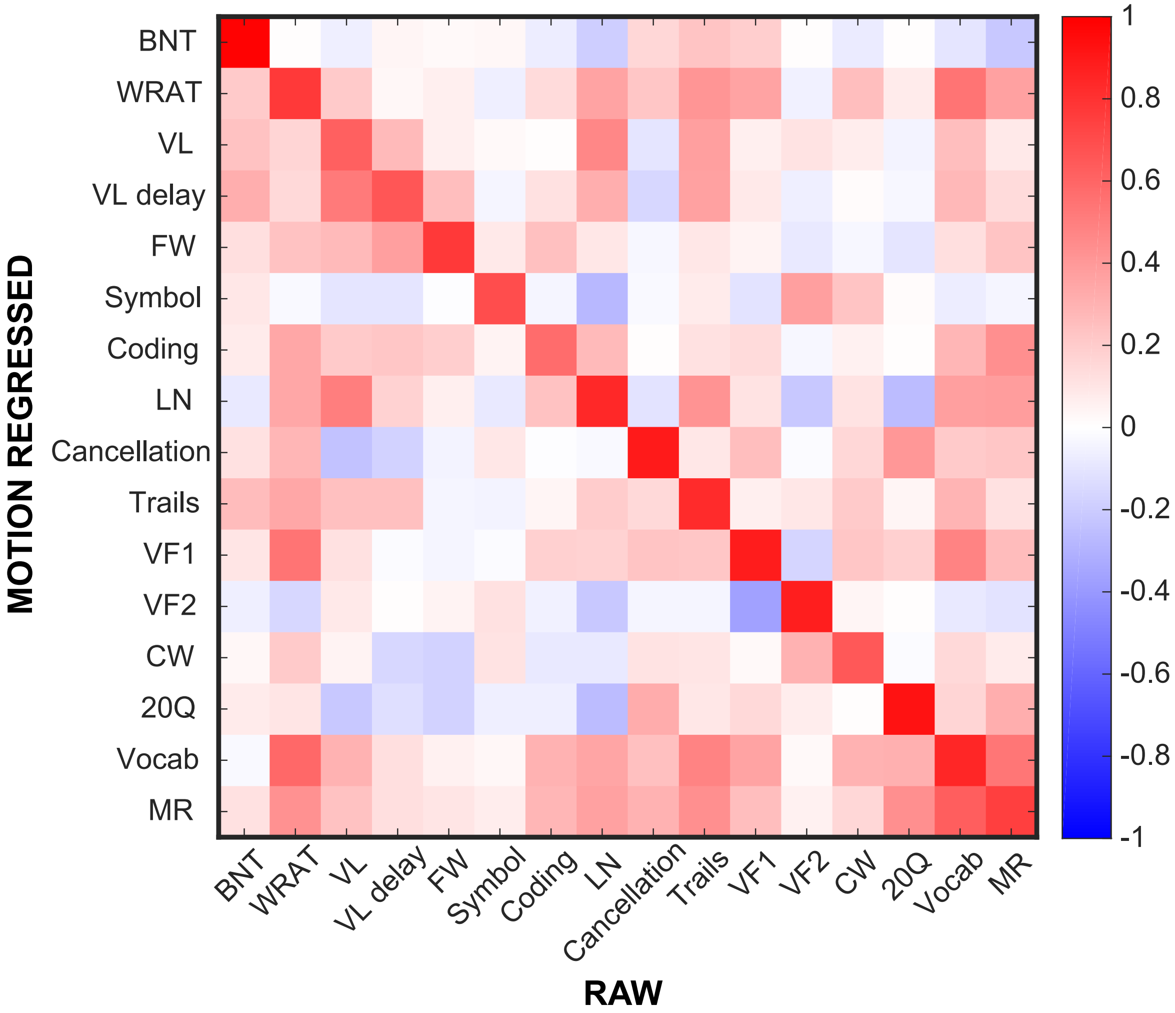

Supplementary Figure 1. Similarity (rank correlation) of misclassification frequency (MF; averaged across in-scanner conditions) derived from classification analyses of the given Yale phenotypic measure using raw and motion-regressed functional connectivity (FC). Diagonal: mean = 0.78, s.d. = 0.12.

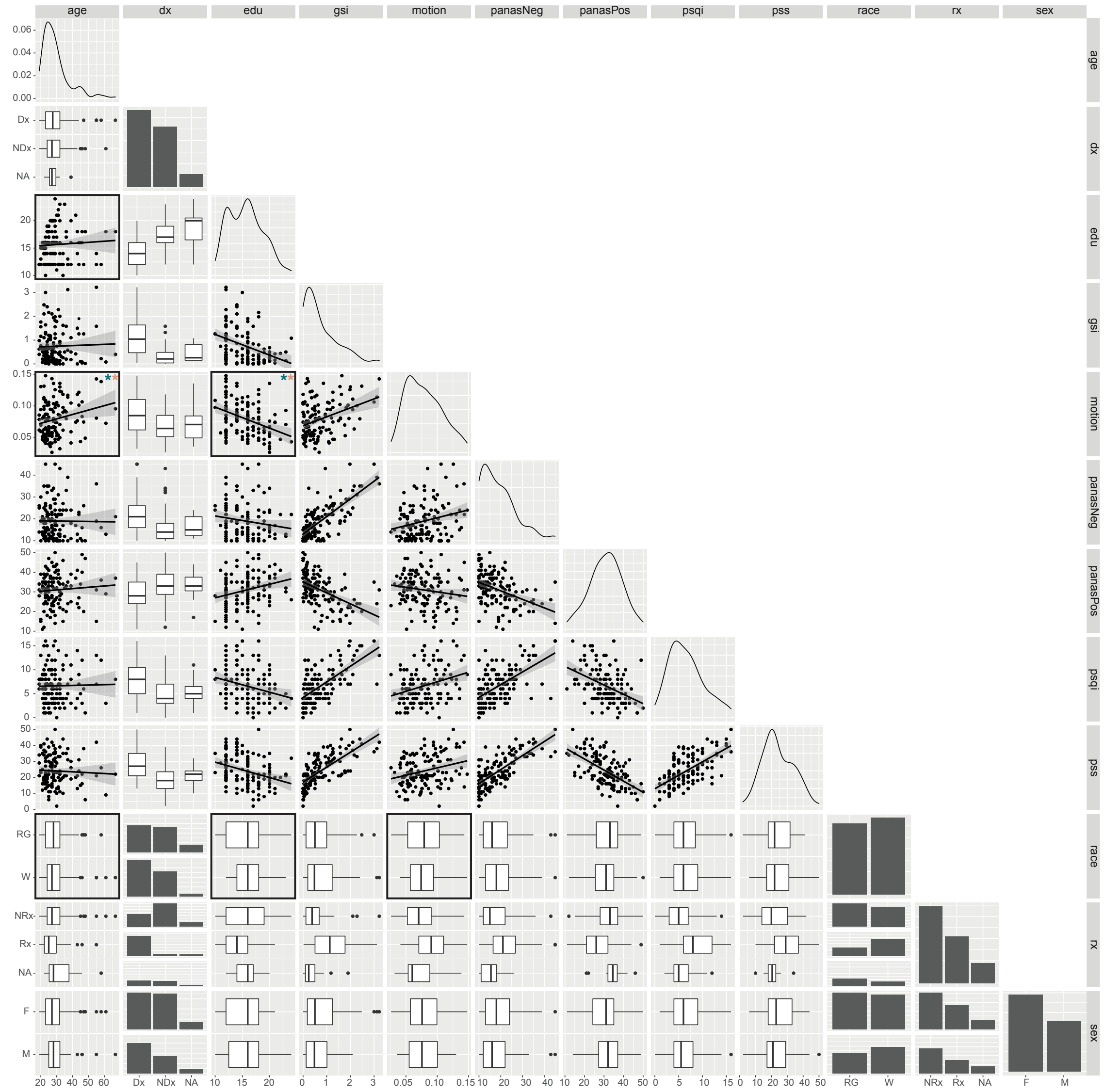

Supplementary Figure 2. Relationships between each pair of covariates in the Yale dataset. Individual variable distributions on the main diagonal. For pairwise relationships, two continuous covariates presented as a scatterplot, one continuous and one categorical as a boxplot, and two categorical as a faceted bar plot. Boxplot line and hinges represent median and quartiles, respectively; whiskers extend to most extreme non-outliers. Lines on scatterplots reflect smoothed conditional means and their 95% CI. Relationships between variables significantly related to MF enclosed in boxes. For each significant ($P < 0.05$, via two-tailed Spearman correlation and Mann-Whitney U test) such relationship, we re-tested the relationship between each variable in the pair with MF while controlling for the other, either via partial Spearman correlation (continuous covariate of interest, continuous or binary control covariate) or via Mann-Whitney U test (binary covariate of interest and residualized MF [i.e., regressed on continuous control covariate]), both two-tailed. *Significant ($P < 0.05$) and main result-consistent (i.e., sign/direction) relationship with low-scorer MF after control, *significant ($P < 0.05$) and consistent relationship with high-scorer MF after control. No significance test results (i.e., asterisks) reported for covariate pairs that are not significantly associated with each other. Covariate pairwise n = 101-129. For related results, see regression analyses reported in Supplementary Tables 9 and 10. RG, racialized groups; W, white; NRx, not taking psychiatric medication; Rx, taking psychiatric medication; NDx, no diagnosis via interview; Dx, one or more diagnoses via interview; F, female; M, male; gsi, Brief Symptom Inventory global severity index.

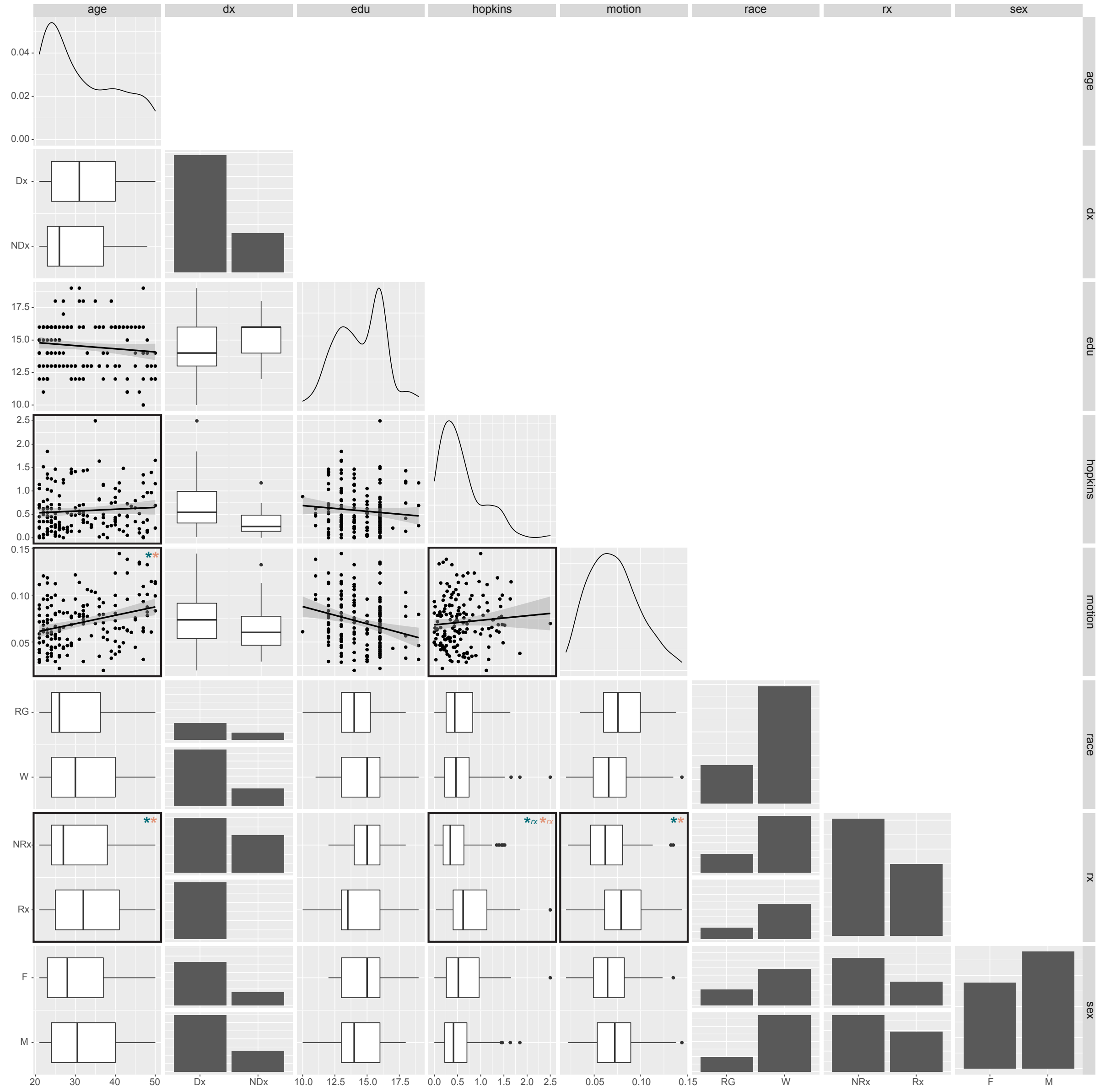

Supplementary Figure 3. Relationships between each pair of covariates in the UCLA dataset, presented as in Supplementary Fig. 2. If only one covariate in pair remained significantly related to MF after control, that covariate's name is reported (e.g., *rx*rx for Hopkins/Rx indicates that medication status remains significantly associated with low-scorer and high-scorer MF after controlling for symptom severity, but symptom severity is not significantly associated with low-scorer or high-scorer MF after controlling for medication status). Covariate pairwise $n = 163$.

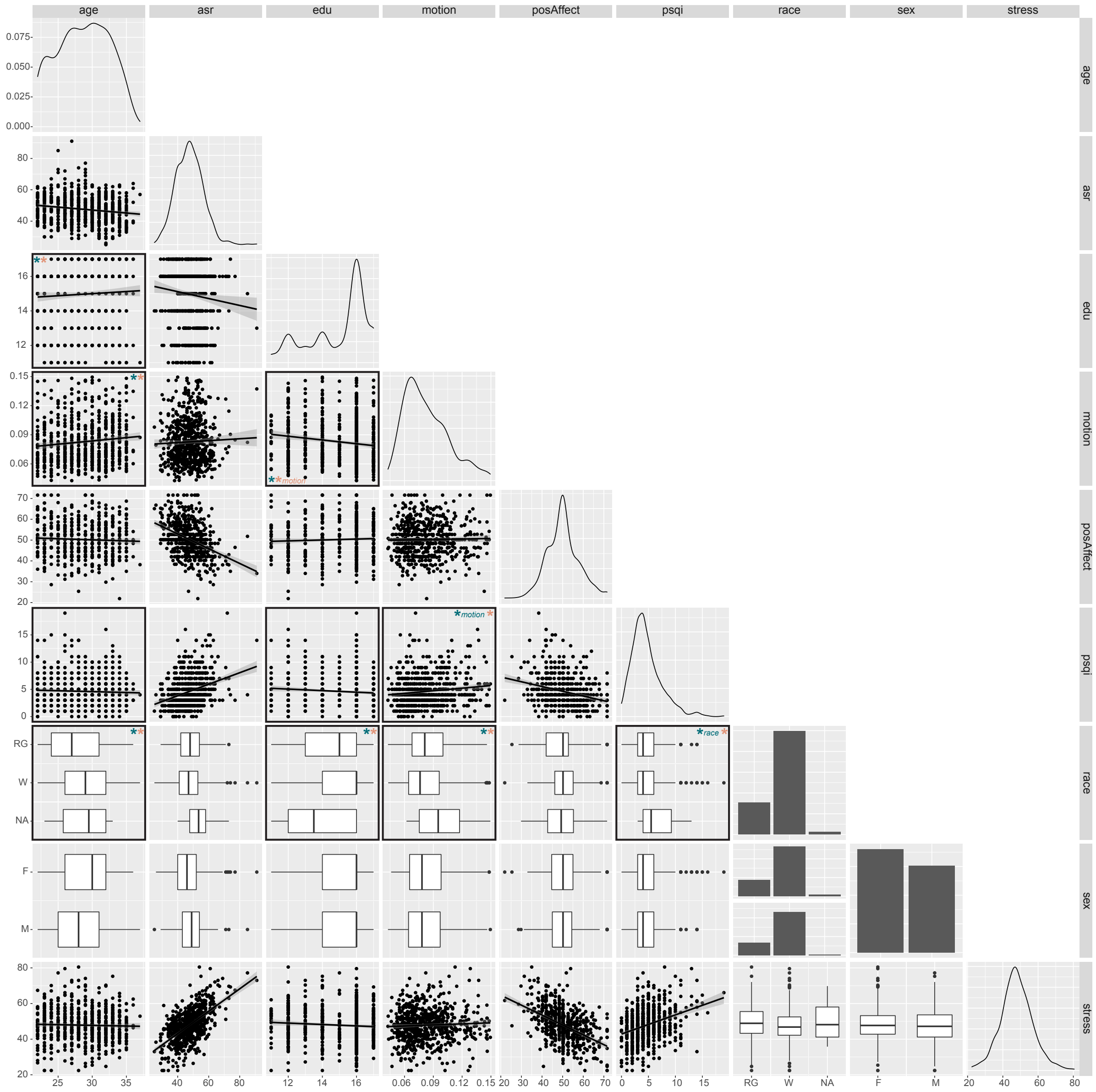

Supplementary Figure 4. Relationships between each pair of covariates in the HCP dataset, presented as in Supplementary Figs. 2-3. Covariate pairwise *n* = 651-664. asr, Achenbach Adult Self-Report.

Supplementary Tables

Task	RDoC Domain	RDoC Construct	Task Description
Card Guessing Task[1,2]	Positive valence	Reward responsiveness	Participants guess whether the number on a card is lower than 5 or greater than 5 but less than 10. Guess accuracy is deterministic: on high-win blocks, participants are correct 70% of the time, and on high-loss blocks, participants are incorrect 70% of the time.
n-back Task[3–6]	Cognitive systems	Working memory	Participants must determine whether an image is the same as or different than the image that came two before (i.e., 2-back, p(target) = 10%).
Stop-Signal Task[7] (SST)	Cognitive systems	Cognitive control	Participants must indicate whether an arrow stimulus is pointing left or right, and must withhold a response on trials when the arrow turns blue (p(signal) = 25%).
Reading the Mind in the Eyes Task[8]	Social processes	Perception and understanding of others	Participants view images of an individual's eyes, and must select the adjective (out of four choices) that best describes what the depicted person is thinking or feeling.
Perceptual Task (movie watching)	Cognitive systems	Perception	Participants watch three movie clips (trailer from Inside Out, marriage scene from The Princess Bride, and trailer from Up), each approximately 2 minutes long.
Sustained Attention Task[9]	Cognitive systems	Attention	Participants respond via button press to images of cities, but withhold response to images of mountains (p(target) = 10%), with gradual transitions between stimuli.

Supplementary Table 1. In-scanner tasks and corresponding RDoC domains and constructs.

Measure	Subtests	RDoC Domain	RDoC Construct
Demographics questionnaire[#]	N/A	N/A	N/A
Handedness inventory[10]	N/A	N/A	N/A
Interpersonal Reactivity Index[11]	N/A	Social processes	Perception and understanding of others
Perceived Stress Scale (PSS)[12#]	N/A	N/A	N/A
Positive and Negative Affect Schedule (PANAS)[13#]	N/A	Positive/negative valence	N/A
Pittsburgh Sleep Quality Index (PSQI)[14#]	N/A	Arousal and regulatory systems	Sleep-wakefulness
Temperament questionnaire[15]	N/A	N/A	N/A
Task strategy/difficulty questionnaire	N/A	N/A	N/A
Delis-Kaplan Executive Function Scale (D-KEFS)[16*]	Verbal Fluency (VF; Letter and Category Fluency)	Cognitive systems	Language
	Trail Making (Number-Letter Switching)	Cognitive systems	Attention, Perception
	Color-Word Interference (CW; Inhibition)	Cognitive systems	Cognitive control
	Twenty Questions (20Q; Weighted Achievement Score)	Social processes	Social communication
Wechsler Adult Intelligence Scale (WAIS)[17*]	Letter-Number Sequencing (LN)	Cognitive systems	Working memory
	Symbol Search	Cognitive systems	Attention, Perception
	Cancellation	Cognitive systems	Attention, Perception
	Coding	Cognitive systems	Attention, Perception
Wechsler Abbreviated Scale of Intelligence (WASI)[18*]	Vocabulary (Vocab)	Cognitive systems	Language
	Matrix Reasoning (MR)	N/A	N/A
Wide Range Assessment of Memory and Learning (WRAML)[19*]	Finger Windows (FW)	Cognitive systems	Working memory, Declarative memory

	Verbal Learning (VL; immediate and delay recall)	Cognitive systems	Working memory, Declarative memory
Wide Range Achievement Test (WRAT)[20*]	Reading	N/A	N/A
Behavior Rating Inventory of Executive Function (BRIEF)[21]	N/A	Cognitive systems	Cognitive control
Boston Naming Test (BNT)[22*]	N/A	Cognitive systems	Language
Brief Symptom Inventory (BSI)[23#]	N/A	N/A	N/A
Mini-International Neuropsychiatric Interview (MINI)[24#]	N/A	N/A	N/A

Supplementary Table 2. Measures used in the post-scan behavioral battery, with corresponding RDoC domains and constructs where relevant. *Outcome (i.e., "phenotypic") measures used in classification analyses. [#]Covariates used in post-hoc analyses.

	Yale	UCLA	HCP
**Sex**	F = 78, M = 51	F = 69, M = 94	F = 361, M = 303
**Age**	m = 29.5, s.d. = 8.9, n = 129	m = 31.8, s.d. = 9.1, n = 163	m = 28.7, s.d. = 3.8, n = 664
**Race**	RG = 62, W = 67	RG = 40, W = 123	RG = 154, W = 498
**Symptom severity**	m = 0.74, s.d. = 0.73, n = 129	m = 0.58, s.d. = 0.46, n = 163	m = 47.6, s.d. = 8.7, n = 663
**Education**	m = 15.6, s.d. = 3.1, n = 128	m = 14.5, s.d. = 1.8, n = 163	m = 15.0, s.d. = 1.7, n = 664
**Stress**	m = 24.0, s.d. = 9.6, n = 127		m = 47.8, s.d. = 9.2, n = 663
**PSQI**	m = 6.6, s.d. = 3.7, n = 125		m = 4.6, s.d. = 2.8, n = 664
**Positive affect**	m = 31.0, s.d. = 8.2, n = 128		m = 50.3, s.d. = 7.9, n = 663
**Negative affect**	m = 19.0, s.d. = 8.0, n = 129		
**Rx**	NRx = 69, Rx = 42	NRx = 101, Rx = 62	
**Dx**	NDx = 52, Dx = 66	NDx = 41, Dx = 122	

Supplementary Table 3. Demographic and clinical information for the Yale, UCLA, and HCP datasets. m, mean; s.d., standard deviation. For categorical variables, number of participants per group reported. Note that different measures (and thus scales) were in many cases used across datasets (see main text).

	R1	Card	Eyes	grad	Mov	NB	SST	R2	GFC
**BNT** (85, 46-48)	0.60	0.58	0.61	0.61	0.51	0.65	0.60	0.54	0.62
**WRAT** (108, 64-66)	0.56	0.69*	0.63	0.62	0.70*	0.61	0.64*	0.67*	0.65*
**VL** (117, 88-90)	0.57	0.63*	0.68*	0.63	0.72*	0.71*	0.65*	0.59	0.68*
**VL delay** (117, 80-82)	0.55	0.60	0.64*	0.58	0.66*	0.68*	0.68*	0.61	0.63*
**FW** (113, 64-66)	0.64	0.67*	0.69*	0.67*	0.75*	0.67*	0.78*	0.73*	0.68*
**Symbol** (117, 78-80)	0.53	0.59	0.57	0.54	0.61	0.58	0.52	0.63*	0.57
**Coding** (123, 112-114)	0.52	0.60	0.63	0.62	0.68*	0.66*	0.64	0.65*	0.66*
**LN** (96, 78-80)	0.52	0.59	0.62	0.58	0.67*	0.60	0.63	0.64*	0.63*
**Canc** (109, 74-76)	0.45	0.48	0.45	0.42	0.51	0.43	0.49	0.45	0.43
**Trails** (109, 74-76)	0.41	0.50	0.61	0.51	0.65*	0.57	0.50	0.48	0.50
**VF1** (119, 60-62)	0.46	0.62	0.55	0.59	0.59	0.60	0.60	0.61	0.63*
**VF2** (110, 58-60)	0.47	0.50	0.55	0.50	0.63	0.54	0.57	0.50	0.51
**CW** (106, 56-58)	0.56	0.59	0.60	0.49	0.52	0.60	0.52	0.46	0.57
**20Q** (113, 54-56)	0.49	0.54	0.55	0.58	0.65	0.51	0.54	0.59	0.55
**Vocab** (111, 56-58)	0.52	0.65*	0.69*	0.61	0.74*	0.60	0.62*	0.70*	0.68*
**MR** (94, 56-58)	0.64	0.69*	0.75*	0.69*	0.88*	0.74*	0.77*	0.78*	0.76*

Supplementary Table 4. Mean classification accuracy (averaged across 100 iterations) for each phenotypic measure using FC calculated from all in-scanner conditions in the Yale dataset. Number of classified individuals (i.e., high or low, non-outlier score) for each measure, followed by size of training sample (range given subsampling after holding out test data) in parentheses. Canc, cancellation; Mov, movies; NB, *n*-Back. *$P = 0.03$, FDR adjusted (144 comparisons) via one-tailed permutation testing.

	Rest	BART	PAMe	PAMr	SCAP	SST	TS	GFC
**LN** (112, 98-100)	0.53	0.56	0.56	0.64 (0.03)	0.59	0.63 (0.03)	0.61 (0.04)	0.60
**Vocab** (145, 72-74)	0.56	0.61	0.66 (0.03)	0.66 (0.04)	0.57	0.68 (0.03)	0.64 (0.04)	0.66 (0.04)
**MR** (153, 76-78)	0.54	0.59	0.62	0.64 (0.03)	0.50	0.63 (0.03)	0.58	0.62 (0.03)

Supplementary Table 5. Mean classification accuracy (averaged across 100 iterations) for each phenotypic measure using FC calculated from all in-scanner conditions in the UCLA dataset. Number of classified individuals (i.e., high or low, non-outlier score) for each measure, followed by size of training sample (range given subsampling after holding out test data) in parentheses. BART, balloon analog risk task; PAMe, paired associates memory task – encoding; PAMr, paired associates memory task – retrieval; SCAP, spatial working memory capacity tasks; TS, task switching. Significance determined via one-tailed permutation testing; significant P values ($P < 0.05$) in parentheses, all FDR adjusted (24 comparisons).

	Emo	Gam	Lang	Mot	Rel	Soc	WM	R1	R2	GFC
**cIQ** (548, 184-228)	0.64 (0.001)	0.65 (0.001)	0.7 (0.001)	0.7 (0.001)	0.64 (0.001)	0.64 (0.001)	0.67 (0.001)	0.6 (0.004)	0.6 (0.006)	0.69 (0.001)
**fIQ** (532, 280-340)	0.57 (0.008)	0.61 (0.001)	0.61 (0.001)	0.62 (0.001)	0.57 (0.01)	0.59 (0.001)	0.65 (0.001)	0.6 (0.001)	0.56 (0.03)	0.63 (0.001)

Supplementary Table 6. Mean classification accuracy (averaged across 1000 iterations, given 10-fold analysis) for each phenotypic measure using FC calculated from all in-scanner conditions in the HCP dataset. Number of classified individuals (i.e., high or low score) for each measure, followed by size of training sample (range given subsampling after holding out test data) in parentheses. Significance determined via one-tailed permutation testing; *P* values in parentheses, all FDR adjusted (20 comparisons). Emo, emotion; Gam, gambling; Lang, language; Mot, motor; Rel, relational; Soc, social; WM, working memory (n-Back); R1, rest 1; R2, rest 2.

	BNT	WRAT	VL	VL delay	FW	SS	Coding	LN	Canc	Trails	VF1	VF2	CW	20Q	Vocab	MR
**MoR**	0.59	0.58	0.59	0.54	0.62	0.52	0.54	0.56	0.45	0.52	0.54	0.55	0.54	0.56	0.60	0.68
**Raw**	0.59	0.64	0.65	0.62	0.70	0.57	0.63	0.61	0.46	0.53	0.59	0.53	0.54	0.56	0.65	0.74

Supplementary Table 7. Mean classification accuracy, averaged across iterations and in-scanner conditions for each phenotypic measure in the Yale dataset, using motion-regressed (MoR) and raw FC for classification. SS, Symbol Search. Note that, as expected given that motion, a proxy for performance, has been regressed from the brain data, classification performance is overall lower in the MoR case than in the raw case, further highlighting that phenotypic measures reflect constellations of covariates. This correction is complicated, however, by the group-specific relationships between motion and phenotype (see Causes and implications of model failure).

		Test:	All			CCP			MCP		
		**Train:**	*All*	*CCP*	*MCP*	*All*	*CCP*	*MCP*	*All*	*CCP*	*MCP*
**Yale/UCLA**	**LN**	*Yale train*	0.149	0.309	1.000	0.033	0.033	1.000	1.000	1.000	0.064
		UCLA train	0.079	0.111	1.000	0.033	0.033	1.000	1.000	1.000	0.111
	**MR**	*Yale train*	0.252	0.769	1.000	0.033	0.079	1.000	1.000	1.000	0.166
		UCLA train	0.033	0.079	0.977	0.033	0.033	1.000	0.374	1.000	0.130
	**Voc**	*Yale train*	0.252	0.265	1.000	0.079	0.033	1.000	1.000	1.000	0.033
		UCLA train	0.079	0.096	1.000	0.033	0.033	1.000	1.000	1.000	0.033
**Yale/HCP**	**fIQ**	*Yale train*	0.111	0.096	1.000	0.033	0.033	1.000	1.000	1.000	0.033
		HCP train	0.033	0.033	1.000	0.033	0.033	1.000	1.000	1.000	0.079
	**cIQ**	*Yale train*	0.297	0.243	1.000	0.033	0.033	1.000	1.000	1.000	0.033
		HCP train	0.033	0.033	1.000	0.033	0.033	1.000	1.000	1.000	0.096

Supplementary Table 8. FDR-adjusted (90 tests) *P* values for above-chance performance in cross-dataset analyses, calculated via one-tailed comparison to distribution of accuracy across 100 iterations of classification of permuted labels (respecting family-related limits on exchangeability in the HCP dataset[25,26]). We note that correction for multiple comparisons is complicated by the non-independence of the tests (i.e., within dataset, Test: CCP and MCP are subsets of Test: All), but present FDR-adjusted *P* values as more conservative estimates of significance than uncorrected *P* values. All, whole sample; CCP, correctly classified participants within sample; MCP, misclassified participants within sample. Voc, vocabulary. Yale/UCLA and Yale/HCP indicate the pair of datasets used for presented analyses (e.g., "Yale/UCLA, Yale train" refers to the analysis in which Yale data were used to train the model, which was subsequently tested on UCLA data).

	Yale, low	Yale, high	UCLA, low	UCLA, high	HCP, low	HCP, high
**Sex**	$P > 0.1$	$P > 0.1$	$P > 0.1$	$P > 0.1$	$P > 0.1$	$P > 0.1$
**Age**	$r_s = -0.38$, $P_{FDR} < 0.0001$*	$r_s = 0.32$, $P_{FDR} = 0.001$*	$r_s = -0.46$, $P_{FDR} < 0.0001$*	$r_s = 0.40$, $P_{FDR} < 0.0001$*	$r_s = -0.18$, $P_{FDR} = 0.01$*	$r_s = 0.14$, $P_{FDR} = 0.002$*
**Race**	$median_{RG\text{-}W} = -0.14$, $P_{FDR} = 0.02$*	$median_{RG\text{-}W} = 0.19$, $P_{FDR} = 0.003$*	$P > 0.1$	$median_{RG\text{-}W} = 0.18$, $P = 0.10$	$median_{RG\text{-}W} = -0.29$, $P_{FDR} < 0.0001$*	$median_{RG\text{-}W} = 0.22$, $P_{FDR} < 0.0001$*
**Symptom severity**	$P > 0.1$	$r_s = 0.15$, $P = 0.10$	$P > 0.1$	$r_s = 0.21$, $P_{FDR} = 0.04$	$P > 0.1$	$P > 0.1$
**Education**	$r_s = 0.32$, $P_{FDR} = 0.001$	$r_s = -0.33$, $P_{FDR} = 0.001$	$P > 0.1$	$r_s = -0.17$, $P = 0.05$	$r_s = 0.26$, $P_{FDR} = 0.0002$	$r_s = -0.11$, $P_{FDR} = 0.02$
**Stress**	$P > 0.1$	$P > 0.1$			$r_s = -0.11$, $P = 0.08$	$P > 0.1$
**PSQI**	$P > 0.1$	$P > 0.1$			$r_s = -0.18$, $P_{FDR} = 0.01$	$r_s = 0.16$, $P_{FDR} = 0.0008$*
**Positive affect**	$P > 0.1$	$P > 0.1$			$P > 0.1$	$P > 0.1$
**Negative affect**	$P > 0.1$	$P > 0.1$				
**Rx**	$P > 0.1$	$P > 0.1$	$median_{NRx\text{-}Rx} = 0.35$, $P_{FDR} = 0.0008$*	$median_{NRx\text{-}Rx} = -0.32$, $P_{FDR} = 0.0005$		
**Dx**	$P > 0.1$	$median_{NDx\text{-}Dx} = -0.09$, $P = 0.07$	$median_{NDx\text{-}Dx} = 0.25$, $P = 0.07$	$P > 0.1$		
**PC1 score**	$r_s = 0.31$, $P_{FDR} = 0.007$	$r_s = -0.45$, $P_{FDR} < 0.0001$*	$r_s = 0.29$, $P_{FDR} = 0.02$	$r_s = -0.42$, $P_{FDR} < 0.0001$*	$r_s = 0.33$, $P_{FDR} < 0.0001$	$r_s = -0.20$, $P_{FDR} < 0.0001$*
**PC2 score**	$P > 0.1$	$r_s = 0.2$, $P_{FDR} = 0.11$	$P > 0.1$	$P > 0.1$	$r_s = -0.15$, $P_{FDR} = 0.04$	$r_s = 0.10$, $P_{FDR} = 0.04$*
**PC3 score**	$r_s = -0.21$, $P_{FDR} = 0.10$	$r_s = 0.21$, $P_{FDR} = 0.10$	$P > 0.1$	$P > 0.1$		
**Mean motion**	$r_s = -0.40$, $P_{FDR} < 0.0001$*	$r_s = 0.48$, $P_{FDR} < 0.0001$*	$r_s = -0.51$, $P_{FDR} < 0.0001$*	$r_s = 0.44$, $P_{FDR} < 0.0001$*	$r_s = -0.41$, $P_{FDR} < 0.0001$*	$r_s = 0.40$, $P_{FDR} < 0.0001$*

Supplementary Table 9. Relationships (two-tailed rank correlation for continuous covariates; two-tailed Mann-Whitney U test for binary covariates) between covariates and mean MF, averaged separately for measures on which participants scored low and high. r_s, rank correlation coefficient; P_{FDR}, FDR-adjusted P value across all covariates for the given dataset, presented for uncorrected $P < 0.05$; $median_{X\text{-}Y}$, difference

in medians between groups X and Y; RG, racialized groups; W, white; NRx, not taking psychiatric medication; Rx, taking psychiatric medication; NDx, no diagnosis via interview; Dx, one or more diagnoses via interview. For clarity and concision, test statistics only reported for $P \leq 0.1$ for a given measure (results with $P > 0.1$ in gray). *Significant ($P < 0.05$) terms in regression of mean (low/high) MF on subset of covariates significantly related to MF in the given dataset (as in Fig. 4a). *n* in each analysis: Yale low pairwise = 97-126, Yale high pairwise = 99-128, Yale low regression = 96, Yale high regression = 98, UCLA low pairwise = 82, UCLA high pairwise = 131-132, UCLA low regression = 82, UCLA high regression = 131, HCP low pairwise = 227-236, HCP high pairwise = 522-532, HCP low regression = 227, HCP high regression = 519.

	Yale, correct	Yale, misclassified	UCLA, correct	UCLA, misclassified	HCP, correct	HCP, misclassified
**Sex**	$P > 0.1$	$P > 0.1$	$P > 0.1$	$P > 0.1$	$P > 0.1$	$P > 0.1$
**Age**	r_s = -0.19, P_{FDR} = 0.09	$P > 0.1$	r_s = -0.41, P_{FDR} < 0.0001*	r_s = 0.22, P_{FDR} = 0.08	$P > 0.1$*	r_s = 0.12, P_{FDR} = 0.10*
**Race**	$median_{RG-W}$ = -0.83, P_{FDR} = 0.01*	$P > 0.1$	$median_{RG-W}$ = -0.78, P_{FDR} = 0.02	$P > 0.1$	$median_{RG-W}$ = -1.44, P_{FDR} < 0.0001*	$median_{RG-W}$ = 0.17, P_{FDR} = 0.09*
**Symptom severity**	r_s = -0.29, P_{FDR} = 0.005	r_s = -0.17, P = 0.06	r_s = -0.17, P_{FDR} = 0.09	$P > 0.1$	$P > 0.1$	$P > 0.1$
**Education**	r_s = 0.48, P_{FDR} < 0.0001*	r_s = 0.20, P_{FDR} = 0.09	r_s = 0.34, P_{FDR} = 0.0002	$P > 0.1$	r_s = 0.40, P_{FDR} < 0.0001*	r_s = 0.12, P_{FDR} = 0.10*
**Stress**	r_s = -0.17, P = 0.05	$P > 0.1$			r_s = -0.13, P_{FDR} = 0.01	$P > 0.1$
**PSQI**	r_s = -0.24, P_{FDR} = 0.03	$P > 0.1$			r_s = -0.09, P_{FDR} = 0.09	$P > 0.1$
**Positive affect**	$P > 0.1$	$P > 0.1$			$P > 0.1$	$P > 0.1$
**Negative affect**	$P > 0.1$	$P > 0.1$				
**Rx**	$median_{NRx-Rx}$ = 0.77, P_{FDR} = 0.12	$P > 0.1$	$median_{NRx-Rx}$ = 1.20, P_{FDR} = 0.003	$P > 0.1$		
**Dx**	$median_{NDx-Dx}$ = 1.06, P_{FDR} =0.003	$P > 0.1$	$P > 0.1$	$P > 0.1$		
**Mean motion**	r_s = -0.50, P_{FDR} < 0.0001*	$P > 0.1$	r_s = -0.38, P_{FDR} < 0.0001*	r_s = 0.30, P_{FDR} = 0.01*	r_s = -0.32, P_{FDR} < 0.0001*	r_s = 0.22, P_{FDR} = 0.001*

Supplementary Table 10. Relationships (two-tailed rank correlation for continuous covariates; two-tailed Mann-Whitney U test for binary covariates) between covariates and mean phenotypic scores, z-scored within measure and averaged across measures on which each participant was frequently (≥50% of iterations and conditions; "misclassified") and infrequently (<50%; "correct") misclassified. r_s, rank correlation coefficient; P_{FDR}, FDR-adjusted P value, presented for uncorrected $P < 0.05$ (note that adjusted P values differ slightly from those in Fig. 4, which were derived using only the presented subset of measures; all measures presented here [and used for multiple comparison correction within dataset] for completeness); $median_{X-Y}$, difference in medians between groups X and Y; RG, racialized groups; W, white; NRx, not taking psychiatric medication; Rx, taking psychiatric medication; NDx, no diagnosis via interview; Dx, one or more diagnoses via interview. For clarity and concision, test statistics only reported for $P \leq 0.1$

for a given measure (results with $P > 0.1$ in gray). *Significant ($P < 0.05$) terms in regression of mean phenotypic score on subset of covariates significantly related to MF (as in Fig. 4b). *n* in each analysis: Yale correct pairwise = 111-129, Yale misclassified pairwise = 105-123, Yale correct regression = 128, Yale misclassified regression = 122, UCLA correct pairwise = 139, UCLA misclassified pairwise = 92, UCLA correct regression = 139, UCLA misclassified regression = 92, HCP correct pairwise = 492-503, HCP misclassified pairwise = 264-271, HCP correct regression = 492, HCP misclassified regression = 264.

	Relationship to mean residualized prediction deviation
**Sex**	$P > 0.1$
**Age**	$r_s = -0.38$, $P_{FDR} < 0.0001$
**Race**	$\text{median}_{\text{RG-W}} = -0.78$, $P_{FDR} = 0.0005$
**Symptom severity**	$P > 0.1$
**Education**	$r_s = 0.29$, $P_{FDR} = 0.003$
**Stress**	$P > 0.1$
**PSQI**	$P > 0.1$
**Positive affect**	$P > 0.1$
**Negative affect**	$P > 0.1$
**Rx**	$P > 0.1$
**Dx**	$P > 0.1$
**PC1 score**	$r_s = 0.30$, $P_{FDR} = 0.007$
**PC2 score**	$r_s = -0.20$, $P_{FDR} = 0.11$
**PC3 score**	$r_s = -0.17$, $P = 0.09$
**Mean motion**	$r_s = -0.39$, $P_{FDR} < 0.0001$

Supplementary Table 11. Relationships between continuous model fit and covariates in the Yale dataset. In this analysis, only low outliers and missing data, not intermediate scores, were excluded (n = 109-129). Continuous phenotypic measures were predicted using FC (see Methods), and the difference between predicted and observed phenotypic score was regressed on phenotypic score. This residualized prediction deviation was averaged across in-scanner conditions, and then z-scored and averaged across phenotypic measures, yielding a single measure of model fit per participant. These mean residualized prediction deviation metrics were then related, via two-tailed rank correlation for continuous covariates and two-tailed Mann-Whitney U test for binary covariates, to the same covariates presented in Supplementary Table 9 (pairwise n = 100-129). As in Supplementary Table 9, FDR-adjusted P values presented for unadjusted $P < 0.05$; test statistics presented for all unadjusted $P \leq 0.1$; all else in gray.

Supplementary Discussion

Model failure as a tool for subtyping

Subtyping, broadly conceived, is by no means a novelty; much work in a range of fields has carved samples into subtypes. For example, many have divided participants by diagnostic criteria and sought neural correlates of these groupings (e.g., melancholic versus non-melancholic major depressive disorder [MDD][27–30], combined, hyperactive-impulsive, and inattentive subtypes of attention-deficit/hyperactivity disorder [ADHD][31–34], and distinct neurodegenerative disorders[35,36]). Others have taken a more data-driven approach. For example, several studies have used behavioral, eye-tracking, or self-report data to group participants and then have compared brain data across resulting subtypes (e.g., in ADHD[37,38], autism[38,39], MDD[40,41], and schizophrenia[42]). Others have reversed this approach, using brain activity to group individuals and then comparing clinical and behavioral measures across subtypes (e.g., in PTSD[43], bipolar disorder[44], and MDD[45,46]). Still others have used principled combinations of brain and phenotypic data to yield subtypes (e.g., in autism[47] and MDD[48,49]). Such work has advanced understanding of clinical heterogeneity and its neural and behavioral correlates, but it is limited by its focus either exclusively on brain or phenotype, or on a single brain-phenotype relationship.

Here, using the relationship between brain and phenotype rather than either alone, we have shown that there is often no single such relationship that holds for all individuals. By identifying correctly classified and misclassified groups for each phenotype, the analytical framework we present offers a new subtyping approach that improves predictive model performance, and thus resolution of brain-phenotype relationships, for a given individual. Identification of such subtypes may permit the parallel development of

large sample-based, broadly generalizable models[50,51], and subtype-specific models that do not require such large samples and that capture meaningfully distinct neural representations of the modeled phenotype. Further, this framework can be extended to consideration of multiple phenotypes, via either joint modeling of multiple phenotypic measures (as in [52]) or post-hoc intersection of phenotype-specific groups to yield more nuanced subtypes. Training models in subgroups, however, may fail to yield useful brain-phenotype relationships if the phenotypic measure itself is biased[53] (see Causes and implications of model failure).

Covariate-outcome relationships may be varied and complex

As discussed in the main text, relationships between covariates and the outcome of interest can take many forms. A phenotypic outcome may be related to a covariate, but this shared variance need not overlap with the variance shared between outcome and brain. Alternatively, it is possible that a given outcome-covariate relationship holds across the entirety of the sample, or that a sample is homogenous in the given domain (e.g., age); in these cases, the covariate would not influence misclassification frequency. Finally, if the outcome-covariate shared variance does overlap with the outcome-brain shared variance[54], it need not do so in the same manner in all participants.

Additional limitations and future directions

In this work, sample constraints permitted us to identify the existence of stereotypical profiles and the implications of profile inconsistency (i.e., misclassification),

but prevented comprehensive, precise profile characterization. In particular, we use the terms “white” and “racialized groups” (see Methods for definition) not to ignore the differences among ethnic, immigrant, cultural, and racial groups in the United States, but rather to address sample size limitations (see Extended Data Fig. 7 for more specific racial and ethnic breakdowns of each sample). While this dichotomy is frequently used in research, we recognize that it is arbitrary, and that race is a crude, often misleading proxy[55] for social and economic disparities (e.g., early-life experiences[56], disparate educational quality[57], socioeconomic status[58], discrimination and perceived discrimination[59], experiences of segregation and racism[60], social status[61], and neighborhood disadvantage[62]). Further, the limitations of using “white” as the de facto reference group[63,64] and education quantity rather than quality[57] have been well documented. We encourage researchers to incorporate these considerations into study design, as well as data collection and analysis (see Causes and implications of model failure: *Limitations and future directions* and Extended Data Fig. 6).

Supplementary references

1. Delgado, M. R., Nystrom, L. E., Fissell, C., Noll, D. C. & Fiez, J. A. Tracking the hemodynamic responses to reward and punishment in the striatum. *J. Neurophysiol.* **84**, 3072–3077 (2000).
2. Speer, M. E., Bhanji, J. P. & Delgado, M. R. Savoring the past: Positive memories evoke value representations in the striatum. *Neuron* **84**, 847–856 (2014).
3. Gevins, A. S. *et al.* Effects of prolonged mental work on functional brain topography. *Electroencephalogr. Clin. Neurophysiol.* **76**, 339–350 (1990).
4. Conley, M. I. *et al.* The racially diverse affective expression (RADIATE) face stimulus set. *Psychiatry Res.* **270**, 1059–1067 (2018).
5. Tottenham, N. *et al.* The NimStim set of facial expressions: Judgments from untrained research participants. *Psychiatry Res.* **168**, 242–249 (2009).
6. Rosenberg, M. D., Finn, E. S., Constable, R. T. & Chun, M. M. Predicting moment-to-moment attentional state. *Neuroimage* **114**, 249–256 (2015).
7. Verbruggen, F., Logan, G. D. & Stevens, M. A. STOP-IT: Windows executable software for the stop-signal paradigm. *Behav. Res. Methods* **40**, 479–483 (2008).
8. Baron-Cohen, S., Jolliffe, T., Mortimore, C. & Robertson, M. Another advanced test of theory of mind: Evidence from very high functioning adults with autism or Asperger Syndrome. *J. Child Psychol. Psychiatry* **38**, 813–822 (1997).
9. Rosenberg, M., Noonan, S., Degutis, J. & Esterman, M. Sustaining visual attention in the face of distraction: A novel gradual-onset continuous performance task. *Atten Percept Psychophys* **75**, 426–439 (2013).
10. Oldfield, R. C. The assessment and analysis of handedness: The Edinburgh inventory. *Neuropsychologia* **9**, 97–113 (1971).
11. Davis, M. H. A multidimensional approach to individual differences in empathy. *J. Pers. Soc. Psychol.* **44**, 113–126 (1983).
12. Cohen, S., Kamarck, T. & Mermelstein, R. A global measure of perceived stress. *J. Health Soc. Behav.* **24**, 385–396 (1983).
13. Watson, D. & Clark, L. The PANAS-X manual for the Positive and Negative Affect Schedule-Expanded Form. *Iowa Res. Online* **277**, 1–27 (1999).
14. Buysse, D. J., Reynolds, C. F., Monk, T. H., Berman, S. R. & Kupfer, D. J. The Pittsburgh Sleep Quality Index: A new instrument for psychiatric practice and research. *Psychiatry Res.* **28**, 193–213 (1989).
15. Evans, D. E. & Rothbart, M. K. Developing a model for adult temperament. *J. Res. Pers.* **41**, 868–888 (2007).
16. Delis, D., Kaplan, E. & Kramer, J. *Delis-Kaplan Executive Function System (D-KEFS)*. (Bloomington, MN: Pearson, 2001).
17. Wechsler, D. *Wechsler Adult Intelligence Scale–Fourth Edition (WAIS–IV).* (Bloomington, MN: Pearson, 2008).
18. Wechsler, D. *Wechsler Abbreviated Scale of Intelligence--Second Edition*. (Bloomington, MN: Pearson, 2011).
19. Sheslow, D. & Adams, W. *Wide Range Assessment of Memory and Learning, Second Edition*. (Bloomington, MN: Pearson, 2003).
20. Wilkinson, G. S. & Robertson, G. J. *Wide Range Achievement Test (WRAT5)*. (Bloomington, MN: Pearson, 2017).
21. Gioia, G. A., Isquith, P. K., Guy, S. C. & Kenworthy, L. Behavior rating inventory of executive function. *Child Neuropsychol.* **6**, 235–238 (2000).
22. Kaplan, E., Goodglass, H. & Weintraub, S. *Boston Naming Test.* (Austin, TX: Pro-Ed, 2001).

23. Derogatis, L. R. *Brief Symptom Inventory*. (Bloomington, MN: Pearson, 1993).
24. Sheehan, D. V *et al.* The Mini-International Neuropsychiatric Interview (M.I.N.I.): The development and validation of a structured diagnostic psychiatric interview for DSM-IV and ICD-10. *J. Clin. Psychiatry* **59 Suppl 20**, 22–33 (1998).
25. Winkler, A. M., Ridgway, G. R., Webster, M. A., Smith, S. M. & Nichols, T. E. Permutation inference for the general linear model. *Neuroimage* **92**, 381–397 (2014).
26. Winkler, A. M., Webster, M. A., Vidaurre, D., Nichols, T. E. & Smith, S. M. Multi-level block permutation. *Neuroimage* **123**, 253–268 (2015).
27. Yan, M. *et al.* Disrupted regional homogeneity in melancholic and non-melancholic major depressive disorder at rest. *Front. Psychiatry* **12**, 618805 (2021).
28. Foti, D., Carlson, J. M., Sauder, C. L. & Proudfit, G. H. Reward dysfunction in major depression: Multimodal neuroimaging evidence for refining the melancholic phenotype. *Neuroimage* **101**, 50–58 (2014).
29. Guo, C. C., Hyett, M. P., Nguyen, V. T., Parker, G. B. & Breakspear, M. J. Distinct neurobiological signatures of brain connectivity in depression subtypes during natural viewing of emotionally salient films. *Psychol. Med.* **46**, 1535–1545 (2016).
30. Workman, C. I. *et al.* Subgenual cingulate–amygdala functional disconnection and vulnerability to melancholic depression. *Neuropsychopharmacology* **41**, 2082–2090 (2016).
31. Sanefuji, M. *et al.* Double-dissociation between the mechanism leading to impulsivity and inattention in Attention Deficit Hyperactivity Disorder: A resting-state functional connectivity study. *Cortex* **86**, 290–302 (2017).
32. Qian, X. *et al.* Large-scale brain functional network topology disruptions underlie symptom heterogeneity in children with attention-deficit/hyperactivity disorder. *NeuroImage Clin.* **21**, 101600 (2019).
33. Orinstein, A. J. & Stevens, M. C. Brain activity in predominantly-inattentive subtype attention-deficit/hyperactivity disorder during an auditory oddball attention task. *Psychiatry Res.* **223**, 121–128 (2014).
34. Edel, M. A. *et al.* Differential reward processing in subtypes of adult attention deficit hyperactivity disorder. *J. Psychiatr. Res.* **47**, 350–356 (2013).
35. Piggott, M. A. *et al.* Striatal dopaminergic markers in dementia with Lewy bodies, Alzheimer's and Parkinson's diseases: Rostrocaudal distribution. *Brain* **122**, 1449–1468 (1999).
36. Bruun, M. *et al.* Detecting frontotemporal dementia syndromes using MRI biomarkers. *NeuroImage Clin.* **22**, 101711 (2019).
37. Stevens, M. C., Pearlson, G. D., Calhoun, V. D. & Bessette, K. L. Functional neuroimaging evidence for distinct neurobiological pathways in Attention-Deficit/Hyperactivity Disorder. *Biol. Psychiatry Cogn. Neurosci. Neuroimaging* **3**, 675–685 (2018).
38. Vaidya, C. J. *et al.* Data-driven identification of subtypes of executive function across typical development, ADHD, and ASD. *J. Child Psychol. Psychiatry* **61**, 51–61 (2020).
39. Lombardo, M. V *et al.* Default mode-visual network hypoconnectivity in an autism subtype with pronounced social visual engagement difficulties. *Elife* **8**, e47427 (2019).
40. Baller, E. B. *et al.* Neurocognitive and functional heterogeneity in depressed youth. *Neuropsychopharmacology* **46**, 783–790 (2020).
41. Maglanoc, L. A. *et al.* Data-driven clustering reveals a link between symptoms and functional brain connectivity in depression. *Biol. Psychiatry Cogn. Neurosci. Neuroimaging* **4**, 16–26 (2019).
42. Dickinson, D. *et al.* Attacking heterogeneity in schizophrenia by deriving clinical

subgroups from widely available symptom data. *Schizophr. Bull.* **44**, 101–113 (2018).

43. Stout, D. M., Harlé, K. M., Norman, S. B., Simmons, A. N. & Spadoni, A. D. Resting-state connectivity subtype of comorbid PTSD and alcohol use disorder moderates improvement from integrated prolonged exposure therapy in Veterans. *Psychol. Med.* 1–10 (2021). doi:10.1017/S0033291721001513
44. Njau, S. *et al.* Neural subtypes of euthymic bipolar I disorder characterized by emotion regulation circuitry. *Biol. Psychiatry Cogn. Neurosci. Neuroimaging* **5**, 591–600 (2020).
45. Misaki, M., Suzuki, H., Savitz, J., Drevets, W. C. & Bodurka, J. Individual variations in nucleus accumbens responses associated with major depressive disorder symptoms. *Sci. Rep.* **6**, 21227 (2016).
46. Goya-Maldonado, R. *et al.* Dissociating pathomechanisms of depression with fMRI: Bottom-up or top-down dysfunctions of the reward system. *Eur. Arch. Psychiatry Clin. Neurosci.* **265**, 57–66 (2015).
47. Reardon, A. M., Li, K., Langley, J. & Hu, X. P. Subtyping Autism Spectrum Disorder via joint modeling of clinical and connectomic profiles. *Brain Connect.* **12**, 193-205 (2022).
48. Drysdale, A. T. *et al.* Resting-state connectivity biomarkers define neurophysiological subtypes of depression. *Nat. Med.* **23**, 28–38 (2016).
49. Grosenick, L. *et al.* Functional and optogenetic approaches to discovering stable subtype-specific circuit mechanisms in depression. *Biol. Psychiatry Cogn. Neurosci. Neuroimaging* **4**, 554–566 (2019).
50. Benkarim, O. *et al.* The cost of untracked diversity in brain-imaging prediction. *bioRxiv* (2021). doi:10.1101/2021.06.16.448764
51. Lanka, P. *et al.* Supervised machine learning for diagnostic classification from large-scale neuroimaging datasets. *Brain Imaging Behav.* **14**, 2378–2416 (2020).
52. Dubois, J., Galdi, P., Paul, L. K. & Adolphs, R. A distributed brain network predicts general intelligence from resting-state human neuroimaging data. *Philos. Trans. R. Soc. B Biol. Sci.* **373**, 20170284 (2018).
53. Li, J. *et al.* Cross-ethnicity/race generalization failure of behavioral prediction from resting-state functional connectivity. *Sci. Adv.* **8**, 1812 (2022).
54. Dadi, K. *et al.* Population modeling with machine learning can enhance measures of mental health. *GigaScience* **10**, giab071 (2021).
55. Sharpe, R. V. Disaggregating data by race allows for more accurate research. *Nat. Hum. Behav.* **3**, 1240 (2019).
56. Byrd, D. A. *et al.* Early environmental factors, ethnicity, and adult cognitive test performance. *Clin. Neuropsychol.* **20**, 243–260 (2006).
57. Manly, J. J., Jacobs, D. M., Touradji, P., Small, S. A. & Stern, Y. Reading level attenuates differences in neuropsychological test performance between African American and White elders. *J. Int. Neuropsychol. Soc.* **8**, 341–348 (2002).
58. Noble, K. G., McCandliss, B. D. & Farah, M. J. Socioeconomic gradients predict individual differences in neurocognitive abilities. *Dev. Sci.* **10**, 464–480 (2007).
59. Whaley, A. L. Stereotype threat and neuropsychological test performance in the U.S. African American population. *Arch. Clin. Neuropsychol.* **36**, 1361–1366 (2021).
60. Williams, D. R. Race, socioeconomic status, and health. The added effects of racism and discrimination. *Ann. N. Y. Acad. Sci.* **896**, 173–188 (1999).
61. Nguyen, A. W., Hamler, T. C. & Cobb, R. J. Discrimination and chronic kidney disease among Caribbean blacks: The effects of immigration and social status. *Race Soc. Probl.* **10**, 248–258 (2018).
62. Vinopal, K. & Morrissey, T. W. Neighborhood disadvantage and children's cognitive skill trajectories. *Child. Youth Serv. Rev.* 105231 (2020).

63. Henrich, J., Heine, S. J. & Norenzayan, A. The weirdest people in the world? *Behavioral and Brain Sciences* **33**, 61–83 (2010).
64. Johfre, S. S. & Freese, J. Reconsidering the reference category. *Sociol. Methodol.* **51**, 253–269 (2021).
